# Impact of angiotensin receptor blocker product recalls on antihypertensive prescribing in Germany

**DOI:** 10.1038/s41371-020-00425-z

**Published:** 2020-10-14

**Authors:** Ulrike Maria Rudolph, Salka Enners, Marita Kieble, Felix Mahfoud, Michael Böhm, Ulrich Laufs, Martin Schulz

**Affiliations:** 1 Department of Cardiology, University Hospital Leipzig, Leipzig, Germany; 2 German Institute for Drug Use Evaluation, Berlin, Germany; 3 Department of Internal Medicine III–Cardiology, Angiology and Intensive Care Medicine, Saarland University Medical Center, Homburg, Germany; 4 Drug Commission of German Pharmacists, Berlin, Germany; 5 Institute of Pharmacy, Freie Universität Berlin, Berlin, Germany

**Keywords:** Hypertension, Prognosis, Preventive medicine

## Abstract

In Germany, ~8 million patients take angiotensin receptor blockers (ARBs) and 2.25 million of them valsartan. In 2018, contamination of generic ARBs with probable carcinogenic nitrosamines resulted in more than 30 recalls. The impact of such a huge recall has never been explored in Europe. We analyzed the utilization of valsartan, all ARBs, and other alternative antihypertensive drugs in Germany. We used our database of anonymized dispensing data from >80% of community pharmacies at the expense of the statutory health insurance (SHI) funds from January 2017 to December 2019. We analyzed 290.8 million prescriptions, including all oral mono- and fixed-dose combinations of ARBs and plausible alternatives, i.e. ACE inhibitors (ACEi), beta-blockers (BB), and calcium channel blockers (CCB). Utilization was calculated by defined daily doses per 1000 SHI-insured persons per day (DID). Valsartan use decreased substantially after the recalls in July 2018 from 39.0 to 14.2 DID (−64%) in the second quarter of 2019 and to 16.9 DID (−57%) in the fourth quarter of 2019. Simultaneously, the use of alternative ARBs increased from 77.7 DID in the second quarter of 2018 to 121.9 DID (+57%) in the fourth quarter of 2019, mainly due to an increase of candesartan dispensing to 99.8 DID (+73%). There were no changes in the utilization of ACEi, BB, or CCB. The majority of recalled generic valsartan products were replaced by other ARBs, predominantly candesartan, despite documented drug shortages. In contrast to previous safety warnings/recalls, our data do not suggest an under-prescription of antihypertensives during this period.

## Introduction

Angiotensin receptor blockers (ARBs) are the most commonly used drugs for the treatment of arterial hypertension, heart failure, and chronic kidney disease [1]. In Germany, ~8 million patients take ARBs and 2.25 million of them valsartan [2].

In accordance with the *European Medicines Agency*, the *German Federal Institute for Drugs and Medical Devices* announced recalls of generic valsartan products on July 4th, 2018 [3]. The drugs were recalled due to high concentrations of probable carcinogenic nitrosamines found in selected lots of ARB substances and generic drugs, primarily valsartan from *Zhejiang Huahai Pharmaceutical Co., Ltd.*, Linhai, China. These impurities were most likely related to a change in the manufacturing process of the valsartan substance implemented in 2012 [4]. Subsequently, 16 marketing authorization holders recalled affected batches of more than 30 generic drugs containing impurities. Drug products from the originator and a couple of generic products utilizing valsartan from other production facilities were not recalled. Later recalls from August 2018 to March 2019 involved further generic valsartan products as well as generic irbesartan and losartan [5, 6]. Other manufacturers and other drug classes were recognized to be contaminated with nitrosamines and were recalled worldwide [5]. Previous safety warnings and medication recalls were associated with gaps in prescriptions and often associated with adverse health outcomes, also due to drug shortages [7–10].

To the best of our knowledge, the impact of this huge drug product recall on the further use of antihypertensive medication has never been explored in Europe. We, therefore, analyzed the utilization of valsartan, all ARBs, and other alternative antihypertensive drugs, that are angiotensin-converting enzyme inhibitors (ACEi), beta-blockers (BB), and calcium channel blockers (CCB) before and after the recalls in Germany.

## Methods

This drug utilization study used the database of the *German Institute for Drug Use Evaluation* (DAPI) containing anonymous dispensing data of community pharmacies [11]. Data from a representative sample of more than 80% (until 06/2019) and more than 95% (from 07/2019 onwards) of German community pharmacies were available. The data were extrapolated by regional factors to 100% of the population insured by the statutory health insurance (SHI) covering 88% of Germany’s population. Until 06/2019 regional factors were calculated by dividing the number of community pharmacies by the number of pharmacies covered by the DAPI database in the respective region. From 07/2019 onwards, regional factors were calculated by dividing the number of dispensed packages reported by a federal information system about SHI-covered prescriptions known as GAmSi (GKV-Arzneimittel-Schnellinformation) [12] by dispensed packages in the DAPI database in the respective region. Prescriptions for privately insured patients are not covered by our database. Data on the indication, treatment duration, or dosages as well as data on individual patients were not available.

Using the specific product code (*Pharmazentralnummer*, an identification number for pharmaceutical products in Germany), dispensing data were linked to a database containing information on the brand/generic name, composition, active ingredients, package size, dosage form, and route of administration [13].

The dispensings of antihypertensives in Germany in the period from January 2017 to December 2019 were explored to illustrate the changes in utilization after the recalls in the second half of 2018. Apart from ARBs, we analyzed ACEi, BB, and CCB as plausible alternatives as well as for comparison of dispensing data. Hence, all orally administered mono- and fixed-dose combinations containing the following drug classes were included: ARB, ACEi, BB, and CCB.

The allocation of the active ingredients was based on the official version of the *Anatomical Therapeutic Chemical (ATC)* classification system with defined daily doses (DDD) published by the *German Institute of Medical Documentation and Information* [14]. DDD are the assumed average daily maintenance dose for the main indication of a drug in adults [15]. Individual substances were analyzed according to the ATC code level 5. For the analysis of individual ARBs, we included mono preparations and combinations with hydrochlorothiazide only because those were affected by the recalls.

In Germany, antihypertensive drugs are usually dispensed in a 3-months’ supply, that is in general 100 tablets in a norm size N3 drug package. It is possible to prescribe more than one drug package per prescription.

As the drug utilization unit, the *DDD per 1000 inhabitants per day* (DID) was used providing an estimate of the proportion of the population treated daily with a particular drug or group of drugs [15]. The DID is a global, technical unit used for evaluation of drug utilizations and treatment frequencies allowing comparisons across various time periods [16]. Compared to other measurement parameters, DID has the advantage that drug utilization is measured using a standardized amount of active ingredient. To adapt for the SHI-insured population analyzed, we calculated the *DDD per 1000 SHI-insured persons per day*. The numbers of SHI-insured persons were obtained from the *Federal Ministry of Health* [17].

We calculated moving averages per month regarding the respective month and the two previous months to smooth short-term fluctuations and highlight longer-term trends.

For the individual ARBs, we also observed the dispensed DID on a daily basis. To determine differences in the dispensing of 2018 compared with 2017 we divided the DID of the weekdays in June and July 2018, the month of first recalls, by the weekdays in June and July 2017. We excluded Sundays for better comparability.

$${\rm{DID}}\,{\rm{ratio}}\,{\rm{of}}\,{\rm{matched}}\,{\rm{weekdays}} = \frac{{\mathrm{DID}}\left( {2018} \right)_{{\rm{of}}\,{\rm{weekday}}\,{\rm{x}}}}{{\mathrm{DID}}\left( {2017} \right)_{{\rm{of}}\,{\rm{weekday}}\,{\rm{x}}}}.$$

To control for drug expenditures, health insurance funds are closing so-called rebate contracts with, above all, the generic pharmaceutical industry. These contracts require the pharmacist to dispense a specific generic drug product. The inability to fulfill rebate contracts of the SHI in the pharmacy due to unavailability of rebated drugs and the subsequent dispensing of a drug not rebated is documented by printing a specific code on the prescription [18]. The amount of prescriptions with this code was taken as an indicator for potential drug shortages.

## Results

The analyses included 290.8 million prescriptions for antihypertensive drugs from January 2017 to December 2019 (Table 1). 47% of all included product codes of antihypertensive drugs were fixed-dosed combinations with 17% of total dispensings. Of these, 67% were combination products with hydrochlorothiazide with 80% of the dispensings of all combination products, and 49% were combinations containing ARBs and hydrochlorothiazide with 40% of the dispensings of all combination products.

**Table 1:** Number of prescriptions for antihypertensive drugs, dispensed drug packages, and drug packages per prescription 2017–2019.

Year and quarter	AHT prescriptions (million)	Drug packages (million)	Drug packages per prescription
2017 Q1	23.6	28.9	1.22
2017 Q2	23.9	29.4	1.24
2017 Q3	23.6	29.0	1.23
2017 Q4	24.4	30.0	1.23
2018 Q1	23.9	29.4	1.23
2018 Q2	24.4	30.0	1.23
2018 Q3	23.9	29.2	1.22
2018 Q4	24.6	30.2	1.23
2019 Q1	24.0	29.4	1.22
2019 Q2	24.7	30.3	1.22
2019 Q3	24.5	30.1	1.23
2019 Q4	25.2	31.0	1.22
Total	290.8	356.8	1.23
Average per quarter	24.2 million	29.7 million	1.227

*AHT* antihypertensive drugs, *Q* quarter.

**Fig. 1:**
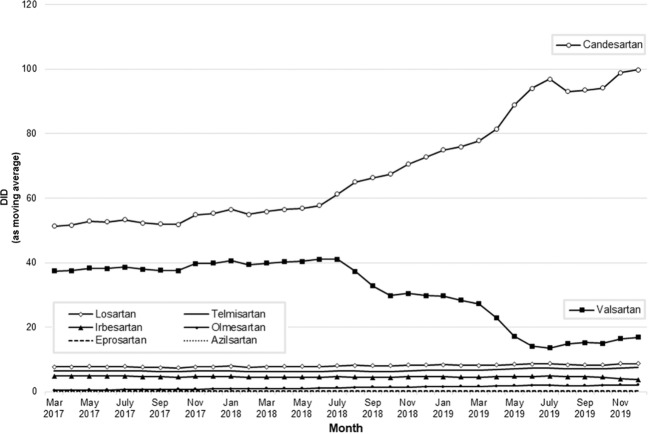
Utilization of angiotensin-II receptor blockers. Utilization of angiotensin-II receptor blockers in DID over time; only mono preparations and fixed-dose combinations with hydrochlorothiazide where included *DID* defined daily doses per 1000 statutory health-insured persons per day.

**Fig. 2:**
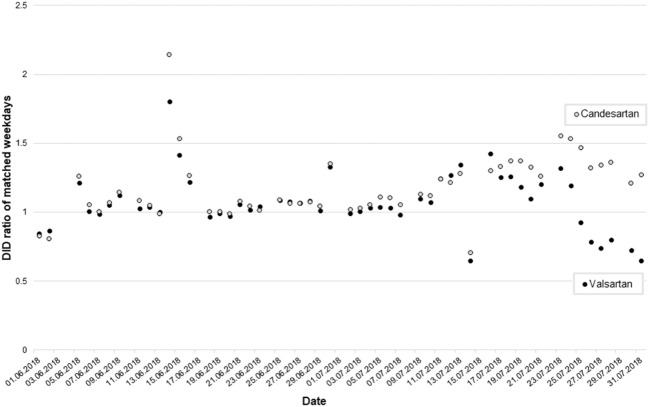
Ratio of the matched daily dispensings for valsartan and candesartan. 2018–2017 ratios of matched weekdays in June and July for dispensings of valsartan and candesartan *DID* defined daily doses per 1000 statutory health-insured persons per day.

The dispensings of valsartan products remained at a constant average level of 39.0 DID from January 2017 until June 2018 (Table 2, Figs. 1 and 2). After the first recalls of valsartan products in July 2018, dispensings decreased substantially and continuously to an average of 27.2 DID (−30%) in the first quarter (Q1) of 2019 and subsequently to 14.2 DID (−64%) in Q2 2019. Throughout the second half of 2019, valsartan dispensings remained low with 15.2 DID (−61%) in Q3 2019 and 16.9 DID (−57%) in Q4 2019 (Table 2; Fig. 1).

**Table 2:** Monthly dispensings of angiotensin receptor blockers in DID from January 2017 to December 2019.

	DID								
Month	Valsartan	Candesartan	Losartan	Telmisartan	Irbesartan	Olmesartan	Eprosartan	Azilsartan	All ARBs
Jan 2017	36.9	50.5	7.7	6.3	4.8	0.4	0.4	0.02	106.9
Feb 2017	36.7	50.4	7.7	6.3	4.7	0.4	0.4	0.01	106.6
Mar 2017	38.6	53.2	7.9	6.6	4.9	0.5	0.4	0.02	112.0
Apr 2017	37.2	51.3	7.6	6.4	4.8	0.5	0.4	0.02	108.2
May 2017	39.1	54.1	7.9	6.6	4.9	0.6	0.4	0.02	113.7
June 2017	38.3	52.6	7.7	6.4	4.8	0.6	0.3	0.02	110.8
July 2017	38.5	53.2	7.7	6.4	4.8	0.7	0.3	0.01	111.6
Aug 2017	37.0	51.0	7.4	6.2	4.5	0.7	0.3	0.01	107.1
Sep 2017	37.5	51.7	7.4	6.2	4.5	0.7	0.3	0.01	108.2
Oct 2017	38.1	52.9	7.5	6.3	4.6	0.8	0.3	0.01	110.4
Nov 2017	43.4	60.1	8.4	7.0	5.1	0.9	0.3	0.02	125.3
Dec 2017	37.9	52.8	7.4	6.2	4.4	0.8	0.3	0.01	109.7
Jan 2018	40.8	56.6	7.9	6.4	4.6	0.9	0.3	0.01	117.5
Feb 2018	39.5	55.4	7.7	6.2	4.5	1.0	0.3	0.02	114.5
Mar 2018	39.3	55.4	7.6	6.2	4.5	1.0	0.3	0.01	114.1
Apr 2018	42.0	58.9	8.1	6.5	4.7	1.1	0.3	0.01	121.5
May 2018	40.1	56.2	7.7	6.1	4.4	1.0	0.3	0.01	115.8
June 2018	41.2	57.9	7.9	6.3	4.5	1.1	0.3	0.01	119.1
July 2018	41.8	69.6	8.5	6.8	4.8	1.4	0.3	0.01	133.3
Aug 2018	28.9	67.5	8.0	6.3	4.4	1.4	0.3	0.01	116.7
Sep 2018	27.4	62.0	7.5	5.9	4.1	1.3	0.3	0.01	108.4
Oct 2018	33.2	73.0	8.5	6.8	4.8	1.5	0.3	0.01	127.9
Nov 2018	30.8	76.7	8.8	7.0	4.8	1.6	0.3	0.01	130.0
Dec 2018	25.5	68.7	7.7	6.1	4.2	1.5	0.2	0.01	113.8
Jan 2019	32.8	79.4	8.6	6.9	4.8	1.7	0.3	0.01	134.4
Feb 2019	26.9	79.7	8.3	6.9	4.6	1.7	0.3	0.01	128.3
Mar 2019	22.1	74.3	7.6	6.3	4.2	1.6	0.2	0.01	116.5
Apr 2019	19.6	90.5	8.8	7.4	4.9	1.9	0.3	0.01	133.3
May 2019	10.0	101.6	8.9	7.6	4.9	2.1	0.2	0.01	135.3
June 2019	13.0	90.0	8.0	6.9	4.5	1.8	0.2	0.01	124.4
July 2019	17.6	99.2	9.1	7.7	5.0	1.9	0.3	0.01	140.8
Aug 2019	13.9	90.0	7.9	6.7	4.4	1.9	0.2	0.01	125.0
Sep 2019	13.9	91.3	7.9	6.8	4.4	1.9	0.2	0.01	126.4
Oct 2019	17.2	101.0	9.1	7.6	4.6	2.1	0.2	0.01	141.8
Nov 2019	18.1	104.4	9.0	7.8	2.9	2.2	0.2	0.01	144.6
Dec 2019	15.5	93.9	8.0	7.0	3.6	2.0	0.2	0.01	130.1

*ARB* angiotensin receptor blocker, *DID* defined daily doses per 1000 statutory health insurance-insured persons per day.

The market shares for generic valsartan products recalled in July and November 2018 were both 45% in the 3-month period prior to the respective recall. Before the first recall, non-recalled and recalled valsartan products together had a market share of 35% of all ARBs, which decreased to 26% (−26%) in the second half of 2018 and to 12% (−66%) in the second half of 2019.

With the decrease of valsartan, alternative ARBs increased by an average of 7.4 DID per quarter (from 77.7 DID in Q2 2018 to 121.9 DID (+57%) in Q4 2019). Candesartan was the ARB with the largest increase in prescription (average increase of 7.0 DID per quarter from 57.7 DID in Q2 2018 to 99.8 DID (+73%) in Q4 2019 (Fig. 1 and Table 2)). For telmisartan, losartan, and olmesartan, only a slight increase of dispensing was observed (Table 2).

Before the first recalls in July 2018, the ratio of the matched daily dispensings for valsartan and candesartan predominantly showed values close to 1, with the exception of public holidays. After the first recalls were instituted, the ratio initially increased to 1.43 for valsartan and 1.30 for candesartan, respectively. Afterwards, the ratio decreased substantially to 0.65 for valsartan while the values for candesartan reached 1.56–1.27 (Fig. 2).

**Fig. 3:**
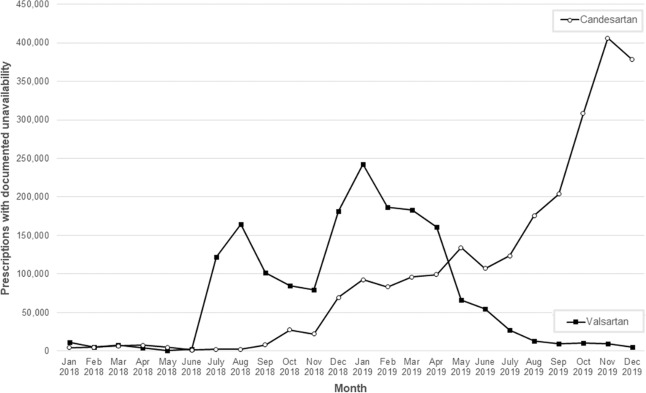
Unavailability of valsartan and candesartan drug products. Number of prescriptions with documented unavailability for valsartan and candesartan drug products over time; only mono preparations and fixed-dose combinations with hydrochlorothiazide were included.

For valsartan and candesartan mono and hydrochlorothiazide combinations, a substantial increase in the amount of prescriptions with documented unavailability of rebated drugs could be observed from July 2018 onwards (Fig. 3). For valsartan, the amount of documented unavailability increased from 2200 prescriptions in June 2018 to a peak of 242,400 prescriptions in January 2019 and subsequently decreased to 4900 in December 2019. For candesartan, the number increased steadily from 1400 in June 2018 to 406,100 in November 2019. In 2019, in total 2.2 million out of 14.9 million (15%) dispensing processes of candesartan drug products were unavailable. For valsartan, in about 1.0 million out of 3.5 million (29%) dispensings, drug products were unavailable.

**Fig. 4:**
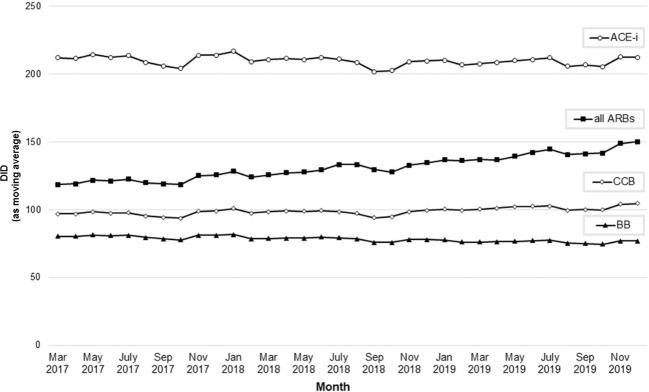
Utilization of antihypertensive drugs. Utilization of antihypertensive drugs in DID over time. ACEi angiotensin-converting enzyme inhibitors, ARB angiotensin-II receptor blockers, BB beta blockers, CCB calcium channel blockers, DID defined daily doses per 1000 statutory health-insured persons per day.

There was no impact of the recalls on the moderate, but continuous increase of the total dispensing of all ARBs. Utilization of ACEi, BB, and CCB remained nearly unchanged (Fig. 4).

## Discussion

The more than 30 recalls of generic drug products containing ARBs from July 2018 to March 2019 due to impurities with probable carcinogenic nitrosamines led to a rapid and substantial decrease in valsartan and a compensatory increase in candesartan dispensings, resulting in an increase in the total volume of ARB prescriptions from 2018 to 2019. Dispensings of ACEi, BB, and CCB remained nearly unchanged. The data, therefore, suggest that patients were primarily switched within the class of ARBs, mostly from valsartan to candesartan. Interestingly, and in contrast to other medication safety warnings/recalls and subsequent drug shortages, the data do not suggest an under-treatment on a population level during this period [7–10, 19].

Valsartan is a guideline-recommended drug for the treatment of hypertension and heart failure and is administered in cumulative daily doses of 80–320 mg [20, 21]. Valsartan was patent-registered in 1991 and became generic in 2011. The market share of generic valsartan before the recalls was 35% of all ARB in Germany. Prior to the generic valsartan recalls, candesartan was already the most frequently prescribed ARB with 57.7 DID, followed by valsartan with 39.0 DID. In contrast, in the US, where losartan is traditionally the most frequently used ARB, recalled generic valsartan was primarily replaced by losartan [22, 23]. Differences in the preferred active pharmaceutical ingredient are possibly influenced by the pharmaceutical company that had initially provided the brand marks in the 1990th or due to drug prices [24].

Our analyses suggests that only a minority of patients received new prescriptions for other ARBs than candesartan, such as losartan, telmisartan, irbesartan, olmesartan, azilsartan, or eprosartan. Similarly, the utilization of plausible alternative drugs remained unchanged suggesting that patients treated with valsartan were switched to another ARB. A change from valsartan to candesartan has the advantage of an easy estimation of the equivalent dose of candesartan (8–32 mg per day) which is roughly one-tenth of the valsartan dose. Physicians seem to prefer a change to an ARB with which they have the most experiences and/or which is broadly available on the market. Within-class replacement of valsartan and the increase of ARB-prescriptions in general seen in our study and the US suggest that physicians are convinced of the efficacy and tolerability of ARBs [21, 23–25].

After the first recall, utilization of valsartan did not decrease by the recalled 45%, but by 28%. About 40% of recalled valsartan might have been replaced by non-recalled valsartan. The second recall, however, led to a reduction of valsartan dispensing that exceeded the amount of the recalled products by ~22%. The intensified decline after several recalls and the reduction of valsartan market share of all ARBs may reflect emerging issues of drug supply and potential uncertainty with regard to additional recalls.

Recently, two studies investigated the consequences of ARB recalls in North-America [19, 23]. Analyzing a database in Ontario, Canada, covering 55,461 patients, 73.8% of recalled valsartan users switched to non-valsartan ARBs and 8.8% to a non-recalled valsartan product within one month after the recall, but 10.7% of patients did not fill an alternative medication at 3 month after the recall [19]. The lack of replacement of recalled valsartan was associated with increased healthcare utilization [19].

In a second study on 9 million generic ARB prescriptions in the United States, 37% of total ARB use prior to recalls was affected by recalls [23]. Generic valsartan use decreased by 53 to 10% in March 2019. Use of losartan increased after the first valsartan recalls from 67 to 73%, but decreased to 71% in March 2019 following recalls of losartan [23].

These as well as our data do not allow conclusions on medication adherence or clinical outcomes. The observed slight increase of prescriptions for antihypertensives in our observation period makes it likely that the majority of patients received new prescriptions. In Germany, the health insurance companies covered the additional costs of new prescriptions of recalled or exchanged nonavailable rebated drugs.

The valsartan recalls led to unavailability of a large amount of rebated valsartan products which was followed by unavailability of rebated candesartan products [26]. It appears that these supply bottlenecks could be substituted by other generic or non-rebated drugs at least within the same quarter. In 2019, valsartan and candesartan were documented in Germany in the group of the ten most active ingredients with unavailability of rebated drugs [26]. As dispensed drug packages of ARBs rose from 2017 to 2019, it might be assumed that this did not result in a shortage of suitable ARBs in general. However, evaluation of unavailable rebated drugs has several pitfalls leading to an underestimation of the entirety of unavailable drugs [26]. For example, only those prescriptions can be counted that have been converted in the pharmacy. Drug shortages are a complex problem of growing concern and represent a constant challenge for pharmacists in everyday practice [8–10].

Two recent trends also affect utilization of cardiovascular drugs: nearly ubiquitous reliance on generic drug products and increased use of manufacturing facilities in China and India [27]. The most common reason for product recalls is manufacturing issues. An investigation of FDA drug recalls lists contamination, mislabeling, adverse reaction, defective product, and incorrect potency as common causes for recalls [28]. Other causes include regulatory issues, discontinuation of products from the market, procurement issues, business decisions and natural disasters [7]. Recalls may trigger errors, confusion and negatively impact medication adherence [29]. Recalls may also impair the relationship of the patient with physicians, pharmacists, and the health care system [30]. Drug recalls may result in change to an active ingredient of second choice, lower dosages, inaccurate use of the alternative medication, a delay or impossibility of a vitally important therapy for patients, or discontinuation of treatment [7–10]. About 60% of pharmacists surveyed in Germany reported impaired medication adherence due to drug shortages [29].

Impurities with nitrosamines, primarily hepatotoxic N-nitrosodimethylamine (NDMA) and N-nitrosodiethylamine (NDEA), may occur in the manufacturing of ARBs containing a tetrazol-group (valsartan, candesartan, irbesartan, losartan, and olmesartan) [22, 31]. On 31th January 2019, the *European Medicines Agency (EMA)* recommended that companies producing ARBs review their manufacturing processes so that nitrosamine impurities do not develop [31]. In humans, nitrosamines were graded by the *World Health Organization* as probable human carcinogens due to their potential to damage DNA. For the vast majority of ARBs, nitrosamine impurities were either not found or were present at very low levels. The extrapolation of the highest possible cancer risk estimated 22 extra cases of cancer due to NDMA over the lifetimes of 100,000 patients who took valsartan from Zhejiang Huahai Pharmaceutical Co., Ltd., China—where the highest levels of impurities were found—every day for 6 years at the highest dose, and eight extra cases in 100,000 patients due to NDEA taking the medicine at the highest dose every day for 4 years. The estimates have been extrapolated from animal studies and are very low compared with the lifetime risk of cancer in the EU (1 in 2) [31]. Anyhow, exposed patients require long-term monitoring [4].

In 2018, a second large AHT class was widely discussed because of potential carcinogenic effects: Hydrochlorothiazide was reported to be dose-dependently associated with development of nonmelanoma skin cancer [32, 33]. Hydrochlorothiazide drug products were not recalled but according to a safety warning via a *Dear Healthcare Professional Letter*, patients had to be informed about the increased risk and the need to control their skin regularly and to use sun protection [34, 35]. This situation likely contributed to the absence of a switch from valsartan to hydrochlorothiazide or thiazide-like diuretics. It also aggravated the overall uncertainty with regard to cardiovascular drug therapies. This is exemplified by the increased reporting of neoplasms [36].

Our study has strengths and limitations. Limitations include not having patient level information including indications for treatment and potential impact on patient outcomes. Some use of ARBs is certainly for indications other than hypertension e.g., heart failure. However, the observed increase of prescriptions of antihypertensive drugs in the observation period makes it likely that most patients received new prescriptions. The strength includes the very large and reliable sample of >290 million prescriptions in ~88% of Germany’s entire population. While for most valsartan drug products all batches were recalled, for some generic products only certain batches were affected by a recall. Due to the impossibility to differentiate between batches in valsartan products, we considered all dispensings of a product as recalled if one batch was affected by the recall. This may have resulted in a small overestimation of recalled generic valsartan but did not change the decrease of overall valsartan use or its share of all ARBs.

## Conclusions

The many recalls of ARBs, primarily generic valsartan drug products from July 2018 to March 2019 due to impurities with probable carcinogenic nitrosamines led to a rapid and substantial decrease in prescriptions for valsartan. Recalled valsartan products were mainly replaced by other ARBs, predominantly candesartan. In addition, the total volume of ARB prescriptions also increased, while utilization of ACEi, BB, and CCB remained nearly unchanged.

Despite the temporal drug shortages of ARBs, our data do not suggest an under-prescribing with antihypertensive drugs during the follow-up period until the end of 2019.

## Summary

### What is known about topic

- In 2018, contamination of angiotensin receptor blockers (ARB) with probable carcinogenic nitrosamines resulted in more than 30 drug recalls.
- In the US, recalled valsartan was mainly replaced by losartan.
- Previous safety warnings and medication recalls were associated with gaps in prescriptions and often associated with adverse health outcomes, also due to drug shortages.
- ARB recalls were associated with increased healthcare utilization in Canada.

### What this study adds

- In Germany, a within class replacement of recalled valsartan products was also found, but mainly to candesartan.
- The total volume of ARB prescriptions increased, while utilization of angiotensin converting enzyme inhibitors, betablockers, and calcium channel blockers remained nearly unchanged.
- Despite temporal drug shortages of ARBs, our data do not suggest an under-prescription with antihypertensive drugs during the follow-up period.
